# Transarterial chemoembolization versus hepatic arterial infusion chemotherapy as first treatment for hepatocellular carcinoma with macrovascular invasion

**DOI:** 10.7150/ijms.108144

**Published:** 2025-02-24

**Authors:** Benyi He, Min Deng, Shaohua Li, Jie Mei, Lianghe Lu, Zhijun Zuo, Rongping Guo, Wei Wei

**Affiliations:** 1 Department of Liver Surgery, Sun Yat-sen University Cancer Center, Guangzhou, 510060, China.; 2 State Key Laboratory of Oncology in South China, Guangdong Provincial Clinical Research Center for Cancer, Sun Yat-sen University Cancer Center, Guangzhou, 510060, China.; 3 Department of General Surgery, The Seventh Affiliated Hospital, Sun Yat-sen University, Shenzhen, 518107, China.; Benyi He and Min Deng contributed to this manuscript equally.

**Keywords:** Hepatocellular carcinoma, Hepatic arterial infusion chemotherapy, Liver, Transarterial chemoembolization

## Abstract

**Introduction:** Transarterial chemoembolization (TACE) is an optional treatment for hepatocellular carcinoma (HCC) patients with macrovascular invasion (MVI) and without extrahepatic metastasis (EHM). As a recently emerging approach, the efficacy of hepatic arterial infusion chemotherapy (HAIC) compared with TACE in this group of patients is unclear.

**Methods:** Between December 2016 and June 2020, patients diagnosed with HCC with MVI and without EHM who underwent TACE (n=91) or HAIC (n=190) as their initial treatment were included. Propensity score matching (PSM) was used to reduce selection bias and other imbalances. The objective response rate (ORR), overall survival (OS), progression-free survival (PFS), rate of subsequent resection, and safety were compared between groups.

**Results:** Seventy-seven pairs of patients were matched after PSM. The ORR was higher in the HAIC group than that in the TACE group (29.9% vs. 9.1%, *P* = 0.013). The median PFS of patients in the HAIC group was longer than that of the patients in the TACE group (4.7 vs. 1.4 months,* P* = 0.002), but there was no significant difference in the median OS between the groups (19.6 vs. 18.1 months, *P* = 0.122). HAIC also showed a better safety profile than TACE.

**Conclusions:** HAIC is an effective and safe option for treating HCC patients with MVI and without EHM compared to TACE.

## Introduction

Hepatocellular carcinoma (HCC) is the sixth most common cancer, fourth leading cause of cancer-related deaths worldwide, and second leading cause of cancer-related deaths in China^1^,^2^. For intermediate-stage HCC, transarterial chemoembolization (TACE) is recommended as the standard treatment in the Barcelona Clinic Liver Cancer (BCLC) staging and the National Comprehensive Cancer Network (NCCN) guidelines 3,4. Nevertheless, approximately 13% to 32% of patients with HCC are diagnosed with portal vein invasion and have a poorer prognosis compared to those without macrovascular invasion (MVI)^5^,^6^. In East Asia, especially the Chinese population, TACE can be used in patients with HCC and MVI^7^. However, serious adverse events (AEs) caused by TACE, such as ectopic embolization, post-embolization syndrome, and abnormal liver and renal functions, mainly relate to the effects of the embolization agents. The presence of MVI may increase the impairment of liver function and further reduce patient tolerance to TACE.

In a retrospective study, hepatic arterial infusion chemotherapy with modified oxaliplatin, fluorouracil, and leucovorin (mFOLFOX-HAIC) improved both progression-free survival (PFS) and overall survival (OS) in patients with advanced HCC treated with sorafenib^8^. When combined with sorafenib, mFOLFOX-HAIC can significantly prolong survival and has a higher tumor response rate (40.8% vs. 2.46%) compared to sorafenib alone, with acceptable AEs rates in patients with HCC and portal vein invasion^9^. In a recent prospective randomized study, HAIC showed significantly superior antitumor efficacy and lower toxicity than TACE in patients with unresectable large HCC^10^.

However, to date, no study has compared the efficacy and safety of TACE and mFOLFOX-HAIC in HCC patients with MVI and without extrahepatic metastasis (EHM). Propensity score matching (PSM) has been proposed to overcome selection bias and increase the level of evidence in retrospective studies. Therefore, this study utilized observational data for PSM to explore the safety and efficacy of mFOLFOX-HAIC and TACE as initial therapies in these patients and to provide the basis for a potential prospective study.

## Patients and methods

### Study design

This retrospective study was conducted at Sun Yat-sen University Cancer Center. The trial protocol was approved by the Institutional Review Board (IRB) and Institutional Ethics Committee (IEC) of Sun Yat-sen University Cancer Center (No. B2018-126-01) and complied with the Declaration of Helsinki. All the patients provided written informed consent to participate in the study. The primary endpoint was OS of patients with MVI and without EHM following TACE or HAIC, while the secondary endpoints were ORR, PFS, rate of subsequent resection, and safety.

### Eligibility criteria

The eligibility criteria for inclusion were as follows: (1) aged≥18 to≤75 years; (2) no previous treatments for HCC; (3) meet the non-invasive diagnosis criteria of the European Association for the Study of Liver^11^ and the American Association for the Study of Liver Diseases^12^; (4) Eastern Cooperative Oncology Group Performance Score (PS)≤2; (5) Child-Pugh score of A or B; (6) The presence of MVI on the image without EHM.

The exclusion criteria were as follows: (1) previous treatment for HCC before TACE or HAIC, (2) HCC combined with other cancers, (3) missing pretreatment or follow-up imaging data, and (4) combined with other antitumor treatments.

**Figure 1:**
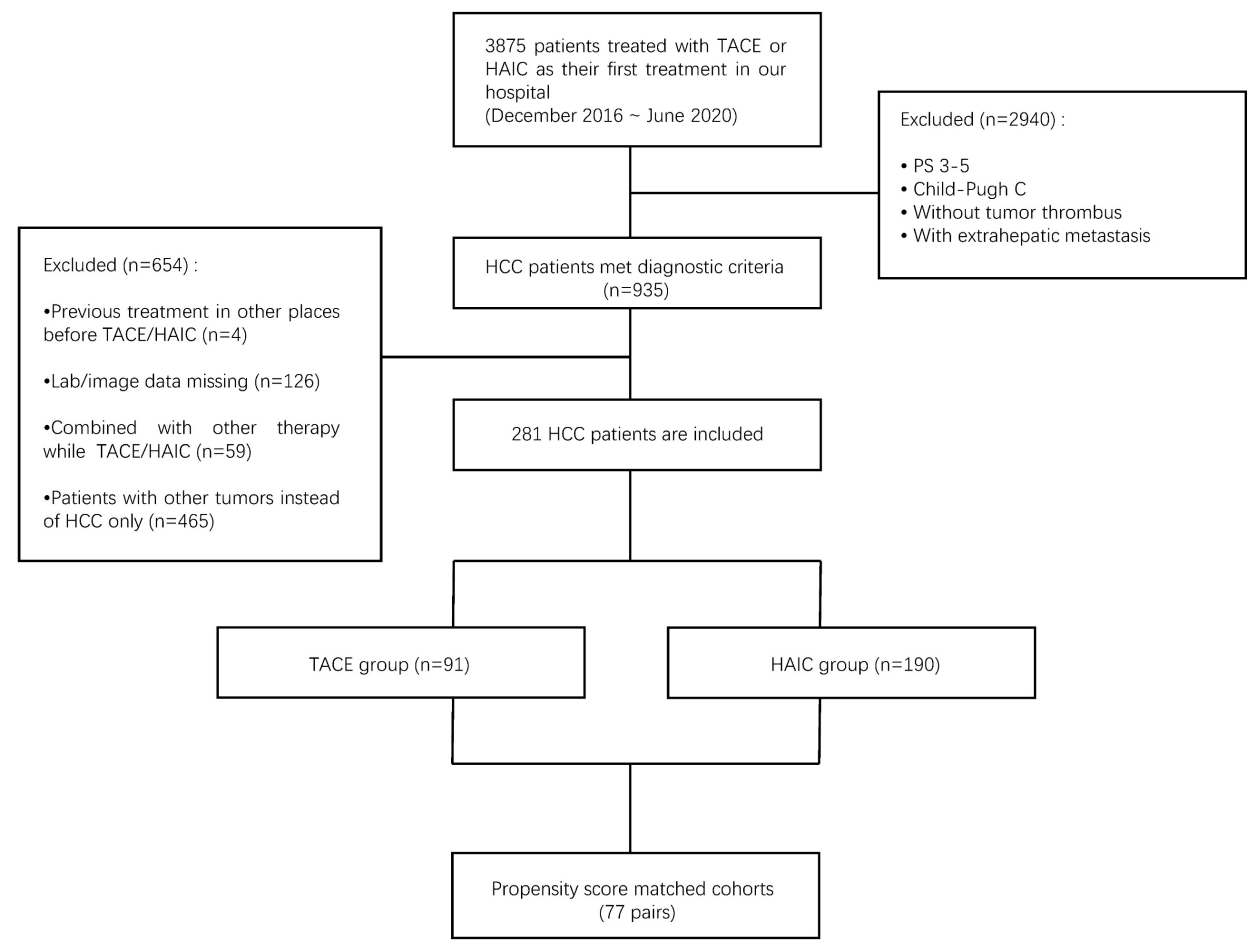
Flowchart of patients selected in the study. HAIC, hepatic artery infusion chemotherapy; TACE, transarterial chemoembolization; HCC, hepatocellular carcinoma.

The detailed process of patient screening is shown in** Figure 1**.

### Treatment procedures

#### Transarterial chemoembolization

TACE was performed using the techniques that we have described previously^13^. Briefly, the catheter was placed into the celiac trunk or superior mesenteric artery for arteriography through the femoral artery using the Seldinger technique, and 50 mg of lobaplatin and 50 mg of epirubicin mixed with iodized oil were injected slowly through the catheter into the feeding arteries of the tumor. Polyvinyl alcohol particles were used to enhance the embolic effect when necessary. TACE was repeated every four weeks until tumor progression or intolerable AEs were observed. Subsequent treatment was determined by the patient's tumor response and opinions from multidisciplinary team discussions.

#### Hepatic arterial infusion chemotherapy

Superior mesenteric and hepatic arteriography were performed after successful percutaneous hepatic artery puncture and catheterization. The main feeding artery of the tumor was then intubated in a predetermined position, and patients with an indwelling catheter were sent back to the ward. The catheter was connected to the injection pump, and the following chemotherapeutic drugs were continuously pumped: oxaliplatin, 130mg/m^2^, from hour 0 to 3 on day 1; leucovorin, 400 mg/m^2^; from hour 3 to 4.5 on day 1; fluorouracil, 400mg/m^2^, from 4.5 to 6.5 on day 1; and fluorouracil, 2400mg/m^2^, over 46 h, from day 1 to day 3. After completion of chemotherapy, the catheter was removed and pressure bandaged to stop bleeding. The patient was discharged after 12 hours. HAIC was repeated every 3 weeks according to the patient's condition. After every two cycles of HAIC, the patients were assessed for tumor response. The subsequent treatment was determined according to the opinions of a multidisciplinary team.

### Follow-up and assessment

All patients were followed up at intervals of 2-3 months. At each scheduled follow-up visit, a physical examination, blood tests (for serum tumor markers and liver function), enhanced abdominal computed tomography, and magnetic resonance imaging were performed. The study will be conducted in July 2022. Tumor response was assessed according to the modified response evaluation criteria in solid tumors (mRECIST) 14, including complete response (CR), partial response (PR), stable disease (SD), and progressive disease (PD). The objective response rate (ORR) was defined as the ratio of patients with CR and PR. The disease control rate (DCR) was defined as the ORR plus SD.

### Statistics analysis

The primary endpoint was OS, defined as the time from initial treatment to the date of death or last follow-up. The secondary endpoint was PFS, which was defined as the time from the initial treatment to the date of tumor progression, death, or last follow-up. TACE- or HAIC-associated AEs were recorded from registration to subsequent treatment, or at the last follow-up. Toxicity was evaluated in accordance with the National Cancer Institute Common Terminology Criteria for Adverse Events (version 5.0)^15^.

Survival curves were estimated using the Kaplan-Meier method and compared using the log-rank test. The median survival with a 95% confidence interval (CI) was calculated. Cox proportional analyses were performed to estimate Hazard Ratio (HR) with 95% CI). The t-test was used for comparisons between groups of continuous variables when they were normally distributed, and the Wilcoxon rank-sum test was used when they were not normally distributed.

Several systemic inflammatory markers, including C-reactive protein (CRP), neutrophil-to-lymphocyte ratio (NLR), platelet-to-lymphocyte ratio (PLR), systemic immune-inflammation index (SII), and prognostic nutritional index (PNI), were analyzed. The SII was determined by multiplying the platelet count by the neutrophil count/lymphocyte count. The PNI was calculated using the following formula: PNI = serum albumin (g/l) + 0.005 × total lymphocyte count (per mm3).

We used propensity score matching (PSM) between the TACE and HAIC groups to reduce the effects of selection bias and potential confounding variables vital to the clinical outcomes. The PSM model included age, sex, Child-Pugh score, cirrhosis, tumor size, number of tumors, Cheng's classification of portal vein tumor thrombus (PVTT)^16^, and TBIL. The number of matched pairs between the TACE and HAIC groups was reduced by 1:1 nearest-neighbor matching with a caliper of 0.2. P values of <0.05 were termed as significant. All statistical analyses were performed using R (version 4.2.1), and graphs were generated using GraphPad Prism 8.

## Results

### Patient characteristics

Between December 2016 and July 2020, 3875 patients with liver tumors underwent TACE or HAIC at our center. Patients who did not meet the inclusion criteria were excluded, including those with PS 3-5, Child-Pugh class C, without MVI, or with EHM (n=2940). For those with previous treatment (n = 4), the imaging data of pretreatment or follow-up were missing (n = 126), other treatments were applied except TACE or HAIC (n = 59), and non-HCC (n = 465) was also excluded. Finally, 281 eligible patients were enrolled in the analysis, including 91 who underwent TACE and 190 who underwent HAIC (**Fig 1**). The baseline patient characteristics are presented in **Table 1**.

Before PSM, the HAIC group differed from the TACE group in terms of cirrhosis (*P* = 0.020) (defined by radiological assessments), tumor size (*P* = 0.012), and total bilirubin (TBIL) (*P* = 0.015). After 1:1 PSM, we obtained matched cohorts of 77 patients per group with well-balanced variables and without significant differences.

### Radiological and clinical response rate

The tumor responses of the patients in the two groups are summarized in **Table 2**. Before PSM, the PR, ORR, and DCR in the HAIC group were 35.3%, 35.3%, and 80.6%, respectively, which were significantly higher than those in the TACE group (8.8%, 8.8%, and 51.7%, respectively). After PSM, the rates of PR, ORR, and DCR in the HAIC group were 29.9%, 29.9%, and 84.4%, respectively, which were still significantly higher than those in the TACE group (9.1%, 9.1%, and 48.1%, respectively).

### Subsequent resection rate

The patients who underwent subsequent resection in the two groups are summarized in **Table 3**. For patients with PVTT of type I-II (according to the Cheng's classification^16^), the subsequent resection rate in the HAIC group was 17.4%, which was significantly higher than that in the TACE group (5.4%; *P* = 0.045). Among patients with type III-IV PVTT, one patient from each of the two groups underwent subsequent resection (*P* = 0.514). After PSM, there was no significant difference between the TACE and HAIC groups in the subsequent resection rates for patients with PVTT of type I-II (*P* = 0.195) and type Ⅲ-Ⅳ (*P* = 1.000).

### OS and PFS analysis

**Figure 2:**
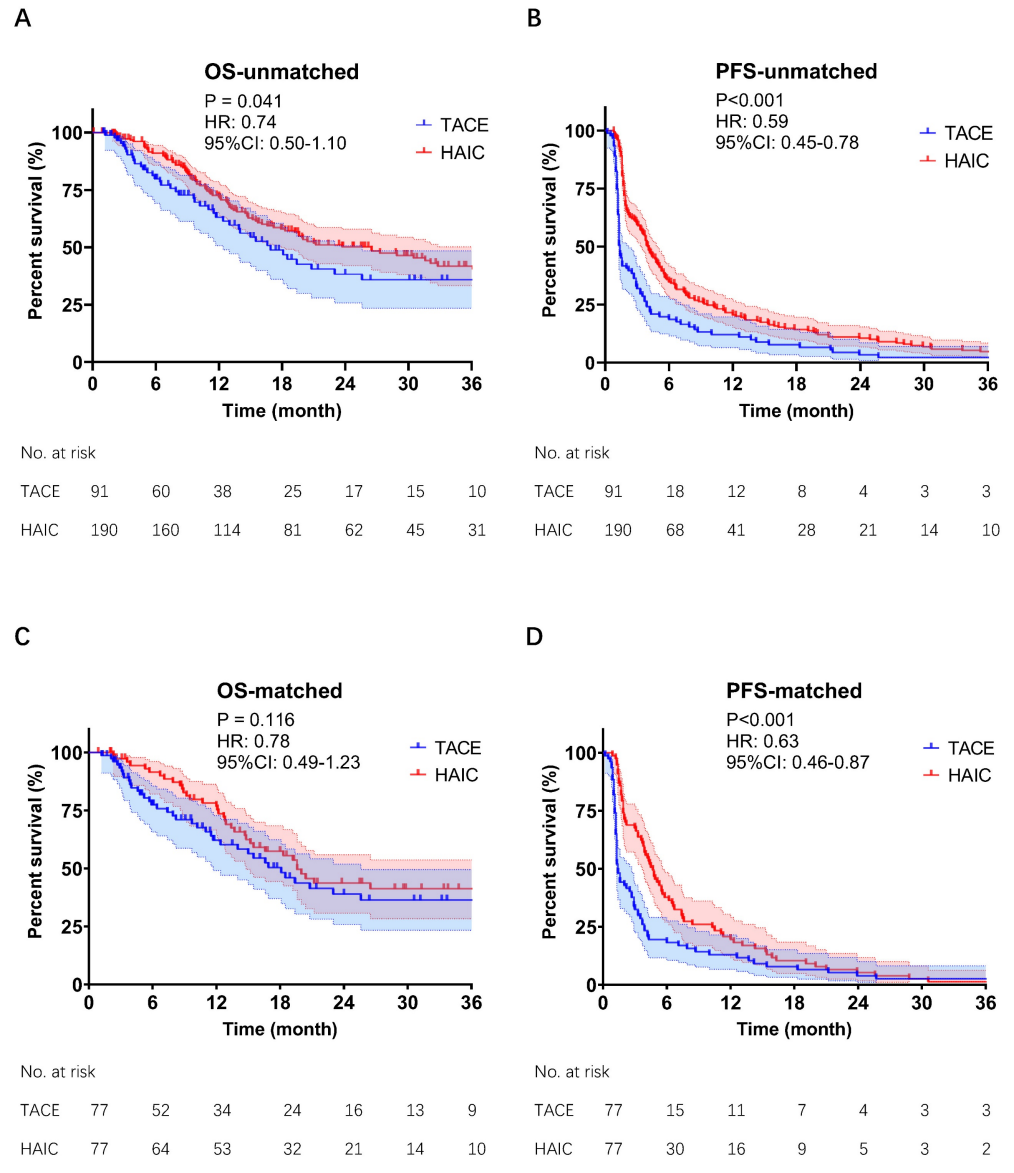
Before (A,B) & after (C,D) PSM of Kaplan-Meier analyses about OS and PFS. TACE group & HAIC group, HCC patients with MVI and without EHM were treated with TACE or mFOLFOX-HAIC as their first treatment. TACE, transarterial chemoembolization; mFOLFOX-HAIC, hepatic arterial infusion chemotherapy of modified fluorouracil, leucovorin, and oxaliplatin; PSM, propensity score matching; OS, overall survival; PFS, progression-free survival; CI, confidence interval; HR, hazard ratio.

The follow-up period ended in July 2022. The incidence of death was 45.1% (41/91) in the TACE group and 46.3% (88/190) in the HAIC group. Patients in the HAIC group had a median OS of 26.5 months compared to 16.9 months in the TACE group (*P* = 0.041, **Fig 2A**). The PFS in the HAIC group was also significantly longer than that in the TACE group (4.1 vs. 1.4 months, *P* < 0.001;** Fig 2C**). After PSM, there was no significant difference in OS between the HAIC and TACE groups (19.6 vs. 18.1 months,* P* = 0.116,** Fig 2B**). However, the PFS in the HAIC group was still significantly longer than that in the TACE group (4.7 vs. 1.4 months, *P* < 0.001,** Fig 2D**). Subgroup analysis of OS showed that the subgroup with preoperative AFP <400 ng/mL benefited most from HAIC (**Fig S1**).

### Adverse events

The treatment-related AEs in the two groups after PSM are summarized in **Table 4**. Overall, adverse reactions were similar and mostly mild in both groups. Compared to the HAIC group, gastrointestinal events such as abdominal pain (19.5% vs. 5.2%, *P* = 0.032) and anorexia (18.2% vs. 3.9%, *P* = 0.023) were more frequent in the TACE group. In addition, one patient in the HAIC group developed flushing, one patient developed ascites, and two patients developed typhlitis. No treatment-related deaths occurred in either group. All of the treatment-related AEs that were observed in the two groups are summarized in **Table S3**.

## Discussion

In this retrospective cohort study, we compared the efficacy and safety of mFOLFOX-HAIC and TACE in propensity score-matched HCC patients with MVI and without EHM. The results showed that mFOLFOX-HAIC significantly improved response rate and PFS. Compared with TACE, mFOLFOX-HAIC resulted in fewer serious AEs. Although there was no significant improvement in the OS and conversion rates for patients in the mFOLFOX-HAIC group, prospective studies using a combination of mFOLFOX-HAIC and other approaches are warranted.

The optimal treatment modalities for patients with macrovascular invasion (corresponding to BCLC stage C and Union for International Cancer Control/National Comprehensive Cancer Network stage IIIB) remain controversial. The 2022 BCLC and NCCN recommend systemic antitumor drugs such as atezolizumab-bevacizumab, lenvatinib, and sorafenib as first-line treatments for patients with vascular tumor thrombus without hepatic function deterioration or poor performance status 17. However, tyrosine kinase inhibitors (TKI) cannot be widely used in China because of their low tumor response rates, modest survival advantages, economic factors, and individual differences 18-22. Although therapeutic strategies for patients with HCC with vascular invasion remain controversial, there is growing evidence that local therapies, including transarterial radioembolization (TARE), TACE, and HAIC, are safe and effective. TACE retains its status as a safe and effective therapy for HCC in East Asia and the Western Pacific region, particularly in patients with vascular invasion. This is despite the introduction of TARE as an alternative, which is limited by its high cost and lack of demonstrated OS benefits, as evidenced by its exclusion from the recommendations for HCC with BCLC stage C in the current guidelines. Recently, arterially directed therapies have been shown to be safe in highly selected patients, in the presence of limited portal vein tumor invasion. Several studies have reported that TACE and HAIC, as the main locoregional therapies of HCC, show encouraging survival benefits and are recommended as standard therapies in the Chinese and Japanese guidelines for the diagnosis and treatment of HCC^23^-^26^. However, the therapeutic approach that is more appropriate for patients with HCC and MVI remains unclear.

In this study, we found that the ORR and DCR of the HAIC group were significantly higher than those of the TACE group (29.9% and 84.4% vs. 9.1% and 48.1%, respectively), according to the mRECIST criteria after PSM. Similar findings were observed in a meta-analysis of patients with Vp3-Vp4 PVTT^20^. The better local antitumor effect of HAIC than that of TACE may be partly due to the following reasons. First, HAIC continuously infuses some therapeutic drugs into the target artery, which results in a higher concentration of the drug accumulating in the lesions. Second, the efficacy of TACE is limited by the incomplete deposition of iodide, especially in the presence of an arterial portal fistula, lack of blood supply in large tumors, and reduced use of iodide due to liver dysfunction. Third, prior studies have shown that while TACE effectively obstructs the arterial blood supply, tumors continue to initiate angiogenesis and attempt to establish new vascular networks and thereby receive blood supply from the portal vein. Fourth, TACE was performed every 4-8 weeks, whereas HAIC was performed every three weeks, as a more proactive strategy with tolerable AEs.

In the present study, a higher proportion of patients in the TACE group had hepatic dysfunction and abdominal pain. This may be because these patients already had a portal vein tumor thrombus. When embolization was added to the feeding artery, the liver had an insufficient blood supply, which might have caused abnormal liver function. In particular, embolism-specific adverse events such as hyperbilirubinemia were significantly more frequent in the TACE group than in the HAIC group. A minority of patients in the HAIC group had specific constitutional symptoms, such as flushing and drug-associated typhlitis, due to the more radical locoregional chemotherapy regimens. A lower incidence of liver dysfunction allows patients to undergo more treatment sessions and achieve better curative effects. Notably, this has been confirmed in previous studies 27.

During the treatment of patients with liver cancer, subsequent therapies may be selected after the initial treatment, which might affect the OS and PFS of our study cohort. As summarized in **Table S2**, more than half of patients in both the TACE and HAIC groups underwent subsequent treatment. Subsequent therapy analysis showed that the patients in the two groups received similar second-line treatments. After PSM, more patients underwent subsequent ablation after the initial TACE than after HAIC (*P* = 0.010). This difference may be attributed to the efficacy of the initial treatment and suggests that ablation is an optional replacement therapy after TACE failure or tumor recurrence. Most patients receiving follow-up treatment were administered systemic therapy, primarily TKIs and PD-1/PD-L1 inhibitors. Several patients in both the TACE and HAIC groups underwent subsequent treatment crossover. In clinical practice, these two treatments have complementary effects, and the selective use of subsequent TACE or HAIC remains necessary for patients with tumors that remain unsuitable for surgical resection.

After PSM, the median OS of the HAIC group was higher than that of the TACE group, but the difference was not statistically significant, whereas PFS was significantly higher in the HAIC group than in the TACE group. As shown in **Table S2**, the proportion of resections after HAIC was higher than that after TACE, and there was a trend for the HAIC group to provide an OS benefit to these patients, although this difference also was not statistically significant. With extended follow-up, the OS benefit of HAIC will be further clarified along with the need for future prospective trials. The OS in our study was higher than that reported in an earlier study that compared mFOLFOX-HAIC and TACE in patients with advanced HCC^28^. Our study did not include patients with EHM, and previous studies have shown that the efficacy of HAIC in patients with EHM is relatively poor^29^. Moreover, the efficacy of HAIC in reducing the levels of serum tumor markers (AFP and PIVKA II) in HCC was significantly better than that of TACE alone. Mechanistically, oxaliplatin infusion can alter the immune microenvironment in liver cancer and affect inflammation levels in the body^30^. Analysis of the level of inflammation indicated significant differences in the TACE group after treatment, with an increase in CRP and NLR and a decrease in PNI levels. Patients in the HAIC group had a decreased PLR, SII, and PNI (**Table S1**). The PNI and NLR are prognostic factors for OS in patients with HCC 31; thus, low-grade inflammation results in better survival benefits in HAIC.

Our study had several limitations that should be noted when interpreting our findings. First, this was a retrospective single-center study, which might have led to a selection bias in patient enrollment. PSM was implemented to reduce the effects of selection bias and confounding factors, but the smaller sample size after PSM may still have affected the results. Second, most of the included patients had hepatitis B-related HCC, and our results must be validated in external institutions with patients with different disease backgrounds. Third, with the rapid development of targeted drugs and immunotherapies, studies on TACE or mFOLFOX-HAIC combined with targeted and immunotherapeutic drugs are required to determine the effects of locoregional therapy.

## Conclusions

The results of this study suggest that mFOLFOX-HAIC might be the better initial therapy option for patients with MVI and without EHM.

## Supplementary Material

Supplementary figure and tables.

## Figures and Tables

**Table 1:** Baseline characteristics of patients in TACE and HAIC groups.

Characteristics		Before PSM			After PSM	
	TACE (n=91)	HAIC (n=190)	P	TACE (n=77)	HAIC (n=77)	P
Age(y), mean ± SD	54.41 ± 12.62	52.45 ± 11.25	0.192	55.22 ± 12.88	53.91 ± 11.75	0.510
Sex						
Male	77 (84.6%)	176 (92.6%)	0.059	67 (87.0%)	70 (90.9%)	0.607
Female	14 (15.4%)	14 (7.4%)		10 (13.0%)	7 (9.1%)	
Child-Pugh score						
5	79 (86.8%)	169 (88.9%)	0.188	71 (92.2%)	69 (89.6%)	0.835
6	8 (8.8%)	19 (10.0%)		5 (6.5%)	7 (9.1%)	
7-9	4 (4.4%)	2 (1.1%)		1 (1.3%)	1 (1.3%)	
Hepatitis virus						
Absence	17 (18.7%)	29 (15.3%)	0.613	16 (20.8%)	13 (16.9%)	0.680
HBV	74 (81.3%)	160 (84.2%)		61 (79.2%)	64 (83.1%)	
HCV	NA	1(0.5%)		NA	NA	
AFP (ng/ml)						
<400	38 (41.8%)	60 (31.6%)	0.123	30 (39.0%)	29 (37.7%)	1.000
≥400	53 (58.2%)	130 (68.4%)		47 (61.0%)	48 (62.3%)	
PIVKAⅡ (mAU/ml)						
<40	3 (3.3%)	6 (3.2%)	1.000	2 (2.6%)	0 (0%)	0.497
≥40	88 (96.7%)	184 (96.8%)		75 (97.4%)	77 (100.0%)	
Cirrhosis						
Absence	70 (76.9%)	168 (88.4%)	0.020	65 (84.4%)	62 (80.5%)	0.672
Presence	21 (23.1%)	22 (11.6%)		12 (15.6%)	15 (19.5%)	
Size of tumor (mm), mean ± SD	83.08 ± 33.79	94.74 ± 37.38	0.012	87.32 ± 31.00	86.55 ± 35.40	0.885
Number of tumors						
Multiple	57 (62.6%)	110 (57.9%)	0.530	47 (61.0%)	46 (59.7%)	1
Single	34 (37.4%)	80 (42.1%)		30 (39.0%)	31 (40.3%)	
Cheng's classification						
TypeⅠ	9 (9.9%)	24 (12.6%)	0.537	6 (7.8%)	8 (10.4%)	0.325
TypeⅡ	65 (71.4%)	125 (65.8%)		57 (74.0%)	53 (68.8%)	
TypeⅢ	17 (18.7%)	38 (20.0%)		14 (18.2%)	13 (16.9%)	
TypeⅣ	0 (0.0%)	3 (1.6%)		0 (0.0%)	3 (3.9%)	
Subsequent resection	5 (5.5%)	27 (14.2%)	0.081	5 (6.5%)	10 (13.0%)	0.336
Blood serum test, mean ± SD						
HGB (g/L)	140.59 ± 22.92	145.26 ± 19.09	0.074	143.43 ± 20.48	144.43 ± 19.24	0.755
PLT (10^9/L)	204.31 ± 94.75	215.93 ± 102.46	0.363	215.75 ± 89.99	216.96 ± 113.19	0.942
ALB (g/L)	40.75 ± 4.18	41.03 ± 4.20	0.598	41.16 ± 3.84	41.13 ± 3.76	0.954
ALP (U/L)	136.34 ± 74.88	151.83 ± 109.31	0.223	135.90 ± 70.08	137.86 ± 89.48	0.880
ALT (U/L)	56.27 ± 42.42	60.85 ± 60.16	0.514	54.75 ± 40.07	58.16 ± 60.96	0.682
TBIL (μmol/L)	20.64 ± 21.90	16.41 ± 6.71	0.015	16.73 ± 7.41	16.78 ± 7.00	0.961
CRE (μmol/L)	73.08 ± 15.72	72.59 ± 18.36	0.828	74.12 ± 16.50	74.70 ± 20.61	0.849

Data were compared by using the Chi square test. PSM, propensity score match; TACE, transarterial chemoembolization; HAIC, hepatic arterial infusion chemotherapy; SD, standard deviation; ALBI, albumin-bilirubin; AFP, Alpha-fetoprotein; PIVKAⅡ, Protein Induced by Vitamin K Absence or Antagonist-II; HGB, hemoglobin; PLT, platelet; ALB, albumin; ALP, alkaline phosphatase; ALT, alanine aminotransferase; TBIL, total bilirubin; CRE, creatinine. Cut-off of AFP is 400ng/ml. Cut-off of PIVKAⅡ is 40mAU/ml.

**Table 2:** Tumor response between TACE and HAIC group.

mRECIST		Non-PSM			PSM	
	TACE(n=91) n(%)	HAIC(n=190) n(%)	P	TACE(n=77) n(%)	HAIC(n=77) n(%)	P
CR	0(0)	0(0)	1.000	0(0)	0(0)	1.000
PR	8(8.8)	67(35.3) *	<0.001	7(9.1)	23(29.9) *	0.013
SD	39(42.9)	86(45.3)	0.904	30(39.0)	42(54.5)	0.305
PD	44(48.4) *	37(19.5)	0.001	40(51.9) *	12(15.6)	0.001
ORR	8(8.8)	67(35.3) *	<0.001	7(9.1)	23(29.9) *	0.013
DCR	47(51.7)	153(80.6) *	0.043	37(48.1)	65(84.4) *	0.042

mRECIST, modified Response Evaluation Criteria In Solid Tumors; PSM, propensity score matching; TACE, transarterial chemoembolization; HAIC, hepatic arterial infusion chemotherapy; CR, complete response; PR, partial response; SD, stable disease; PD, progressive disease; ORR, objective response rate; DCR; disease control rate.

**Table 3:** (A) Number and proportion of patients undergoing resection in TACE and HAIC Groups. (B) Number of patients who received resection in TACE or HAIC group (PSM).

(A)						
Type	Type I - II		Type III - IV			
Treatment	TACE (n=91)	HAIC (n=190)		TACE (n=91)	HAIC (n=190)	
	n(%)	n(%)	P	n(%)	n(%)	P
Total	74(81.3)	149(78.4)		17(18.7)	41(21.6)	
Resection	4(5.4)	26(17.4)	0.045	1(5.9)	1(2.4)	0.514
(B)						
Type	Type I - II		Type III - IV			
Treatment	TACE (n=77)	HAIC (n=77)		TACE (n=77)	HAIC (n=77)	
	n(%)	n(%)	P	n(%)	n(%)	P
Total	63(81.8)	61(79.2)		14(18.2)	16(20.8)	
Resection	4(5.4)	10(16.4)	0.195	1(7.1)	0(0.0)	1.000

Type Ⅰ, Ⅱ, Ⅲ, Ⅳ are defined by Cheng's classification of thrombus. Data in parentheses are percentages. Data were compared by using Chi square test or the Fisher's exact test. PSM, propensity score match; TACE, transarterial chemoembolization; HAIC, hepatic arterial infusion chemotherapy.

**Table 4:** Adverse events appeared in TACE and HAIC group after PSM

Event, n(%)		TACE (n=77)			HAIC (n=77)		P value
	Any grade	Grade 1/2	Grade 3/4	Any grade	Grade 1/2	Grade 3/4	Any grade
Blood/bone marrow suppression							
Leukopenia	4 (5.2)	4 (5.2)	0 (0)	10 (13.0)	9 (11.7)	1 (1.3)	0.209
Neutropenia	6 (7.8)	6 (7.8)	0 (0)	12 (15.6)	10 (13.0)	2 (2.6)	0.276
Reduced hemoglobin level	20 (26.0)	19 (24.7)	1 (1.3)	12 (15.6)	12 (15.6)	0 (0)	0.274
Thrombocytopenia	13 (16.9)	12 (15.6)	1 (1.3)	22 (28.6)	17 (22.1)	5 (6.5)	0.235
Constitutional symptom							
Fever	6 (7.8)	6 (7.8)	0 (0)	1 (1.3)	1 (1.3)	0 (0)	0.118
Dermatology/Skin							
Flushing	0 (0)	0 (0)	0 (0)	1 (1.3)	1 (1.3)	0 (0)	NA
Gastrointestinal events							
Abdominal pain	15 (19.5)	14 (18.2)	1 (1.3)	4 (5.2)	3 (3.9)	1 (1.3)	0.032
Abdominal distension	8 (10.4)	8 (10.4)	0 (0)	13 (16.9)	13 (16.9)	0 (0)	0.429
Anorexia	14 (18.2)	14 (18.2)	0 (0)	3 (3.9)	3 (3.9)	0 (0)	0.023
Ascites	0 (0)	0 (0)	0 (0)	1 (1.3)	1 (1.3)	0 (0)	NA
Typhlitis	0 (0)	0 (0)	0 (0)	1 (1.3)	1 (1.3)	0 (0)	NA
Nausea	3 (3.9)	3 (3.9)	0 (0)	2 (2.6)	2 (2.6)	0 (0)	1.000
Vomiting	3 (3.9)	2 (2.6)	1 (1.3)	1 (1.3)	1 (1.3)	0 (0)	0.620
Hepatic funtion							
Elevated ALP	34 (44.2)	34 (44.2)	0 (0)	42 (52.6)	41 (52.1)	1 (0.5)	0.541
Elevated ALT	37 (48.1)	31 (40.3)	6 (7.8)	38 (46.8)	36 (44.7)	2 (2.1)	1.000
Elevated TBIL	27 (35.1)	24 (31.2)	3 (3.9)	26 (26.3)	21 (23.2)	4 (3.2)	1.000
Hypoalbuminemia	44 (57.1)	44 (57.1)	0 (0)	49 (63.2)	49 (63.2)	0 (0)	0.781
Renal/Genitourinary							
Elevated CRE	16 (20.8)	16 (20.8)	0 (0)	15 (16.8)	13 (15.8)	2 (1.1)	1.000

Data in parentheses are percentages. Data were compared by using Chi square test or the Fisher's exact test. Grade 1/2 referred to combination of Grade 1 and Grade 2. Grade 3/4 referred to combination of Grade 3 and Grade 4. ALP, alkaline phosphatase; ALT, alanine aminotransferase; TBIL, total bilirubin; CRE, creatinine. TACE, transarterial chemoembolization; HAIC, hepatic arterial infusion chemotherapy;
