# Supplementary material for: Transarterial chemoembolization versus hepatic arterial infusion chemotherapy as first treatment for hepatocellular carcinoma with macrovascular invasion
Source: Int J Med Sci. 2025 Feb 24;22(6):1415–24. doi: 10.7150/ijms.108144 (PMC11898842; doi:10.7150/ijms.108144)
Supplement: Supplementary file 1 — Supplementary figure and tables. [file ijmsv22p1415s1.pdf]

Subgroup	TACE	HAIC	HR (95% CI)	P
All Patients	77	77	0.77 (0.49 to 1.23)	0.116
Sex				
Male	67	70	0.79 (0.48 to 1.29)	0.1075
Female	10	7	0.41 (0.11 to 1.54)	0.1875
Age				
<=50 yr	28	29	0.62 (0.26 to 1.47)	0.0812
>50 yr	49	48	0.87 (0.50 to 1.52)	0.6338
Child−Pugh score				
=5	71	69	0.73 (0.45 to 1.19)	0.0696
>5	6	8	1.12 (0.19 to 6.73)	0.7199
Hepatitis virus				
Absence	16	13	0.45 (0.17 to 1.22)	0.1157
HBV	61	64	0.83 (0.48 to 1.41)	0.4798
AFP (ng/ml)				
<400	30	29	0.55 (0.25 to 1.20)	**0.0423**
>=400	47	48	0.92 (0.52 to 1.63)	0.5666
Cirrhosis				
Absence	65	65	0.88 (0.54 to 1.45)	0.622
Presence	12	12	0.31 (0.07 to 1.28)	0.1363
Size of tumor (mm)				
<=100	55	51	0.83 (0.48 to 1.45)	0.3869
>100	22	26	0.55 (0.23 to 1.31)	0.0618
Number of tumors				
Multiple	47	46	1.08 (0.61 to 1.92)	0.7035
Single	30	31	0.58 (0.26 to 1.267)	0.1729
Subsequent resection				
Yes	5	10	0.82 (0.06 to 9.915)	0.8794
No	72	67	0.82 (0.51 to 1.315)	0.4129

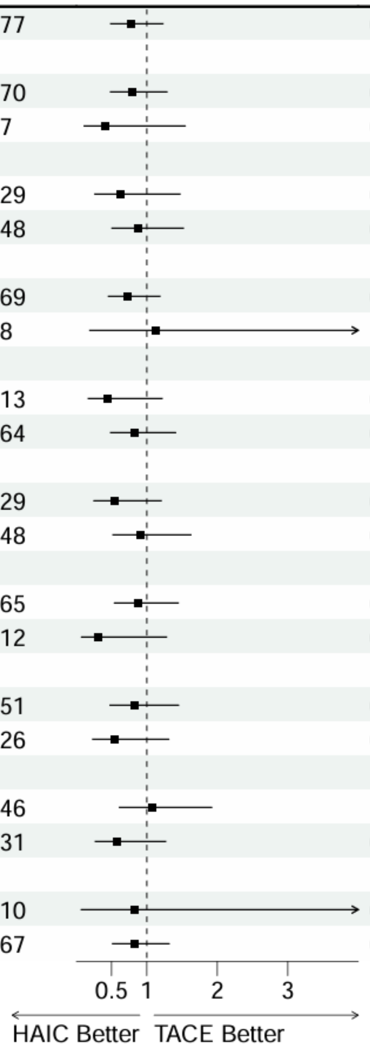

Fig S1. Forest plot for overall survival of patients after propensity score matching analysis. TACE, transarterial chemoembolization; HAIC, hepatic arterial infusion chemotherapy; AFP, Alpha-fetoprotein; HR, hazard ratio; CI, confidence interval.

Supplementary Table 1 | Changes of serum tumor and inflammatory makers between TACE and HAIC group.

Lab findings	TACE (n=91)			HAIC (n=190)		
	**Before**	**After**	**P-value**	**Before**	**After**	**P-value**
**AFP**	32834.8 ± 46618.8	30697.8 ± 43022.6	0.299	31032.1 ± 46919.3	21865.0 ± 43031.8*	<0.001
**PIVKA Ⅱ**	21559.2 ± 28021.2	22170.5 ± 28808.8	0.181	23157.0 ± 28196.5	16996.1 ± 28672.6*	<0.001
**CRP**	17.3 ± 30.5	34.6 ± 42.5*	<0.001	20.7 ± 30.5	21.7 ± 42.6	0.233
**NLR**	2.8 ± 1.8	3.9 ± 14.4*	0.006	3.4 ± 1.8	5.7 ± 14.4	0.202
**PLR**	145.9 ± 90.2	152.8 ± 114.0	0.394	159.9 ± 97.1	136.5 ± 114.0*	<0.001
**SII**	601.5 ± 608.5	706.3 ± 2042.1	0.354	757.6 ± 609.9	843.2 ± 2041.9*	<0.001
**PNI**	48.7 ± 5.6	44.9 ± 7.1*	<0.001	48.6 ± 5.6	45.2 ± 7.0*	<0.001

Changes of serum tumor and inflammatory makers between TACE and HAIC group (PSM)

Lab findings	TACE (n=77)			HAIC (n=77)		
	**Before**	**After**	**P-value**	**Before**	**After**	**P-value**
**AFP**	36020.6 ± 50408.8	34718.3 ± 49458.9	0.246	31817.8 ± 48131.7	25746.6 ± 44440.1*	0.041
**PIVKA Ⅱ**	22779.0 ± 27265.6	23526.8 ± 31314.7	0.181	24151.8 ± 29107.3	16536.8 ± 27118.5*	<0.001
**CRP**	19.0 ± 26.1	36.7 ± 47.6*	<0.001	25.3 ± 43.6	28.4 ± 57.5	0.128
**NLR**	2.9 ± 1.5	3.9 ± 3.8*	0.035	3.4 ± 2.0	5.1 ± 9.5	0.527
**PLR**	154.0 ± 98.1	152.8 ± 137.0	0.394	162.9 ± 109.6	135.3 ± 94.0*	0.020
**SII**	644.1 ± 518.1	747.5 ± 993.9	0.545	780.2 ± 701.7	830.7 ± 1808.6*	0.010
**PNI**	49.3 ± 5.1	45.6 ± 6.7*	<0.001	48.7 ± 5.4	44.5 ± 7.0*	<0.001

AFP, Alpha-fetoprotein; PIVKA Ⅱ, Protein Induced by Vitamin K Absence or Antagonist-II; CRP, C-reactive protein; NLR, Neutrophil-to-lymphocyte ratio, PLR, Platelet-to-lymphocyte ratio; SII, Systemic immune-inflammation index; PNI, prognostic nutritional index; PSM, propensity score matching; TACE, transarterial chemoembolization; HAIC, hepatic arterial infusion chemotherapy.

Data were continuous variables, expressed in (median ± standard deviation). Data in two groups were compared by using the Wilcoxon signed-rank test or matched samples t-test.

Supplementary Table 2 | Number of patients who combined with subsequent treatments in TACE or HAIC group

Treatment	Non-PSM			PSM		
	TACE(n=91)	HAIC(n=190)	P	TACE(n=77)	HAIC(n=77)	P
Total	60	140	0.649	51	58	0.698
Resection	5	27	0.081	5	10	0.336
Ablation	25	15	<0.001	20	5	0.010
Radiotherapy	9	18	1.000	7	7	1.000
Systemic therapy	41	119	0.167	35	50	0.241
TACE	-	38	-	-	15	-
HAIC	16	-	-	14	-	-

PSM, propensity score matching; TACE, transarterial chemoembolization; HAIC, hepatic arterial infusion chemotherapy.

Supplementary Table 3 | Adverse events appeared in TACE and HAIC group

Event, n(%)	TACE (n=91)			HAIC (n=190)		
	**Any grade**	**Grade 1/2**	**Grade 3/4**	**Any grade**	**Grade 1/2**	**Grade 3/4**
**Blood/bone marrow suppression**						
Leukopenia	10 (11.0)	9 (9.9)	1 (1.1)	22 (11.6)	20 (10.5)	2 (1.1)
Neutropenia	9 (9.9)	9 (9.9)	0 (0)	26 (13.7)	20 (10.5)	6 (3.2)
Reduced hemoglobin level	27 (29.7)	25 (27.5)	2 (2.2)	26 (13.7)	26 (13.7)	0 (0)
Thrombocytopenia	22 (24.2)	19 (20.9)	3 (3.3)	53 (27.9)	43 (22.6)	10 (5.3)
**Constitutional symptom**						
Fever	6 (6.6)	6 (6.6)	0 (0)	4 (2.1)	4 (2.1)	0 (0)
**Dermatology/Skin**						
Flushing	0 (0)	0 (0)	0 (0)	1 (0.5)	1 (0.5)	0 (0)
**Gastrointestinal events**						
Abdominal pain	16 (17.6)	15 (16.5)	1 (1.1)	8 (4.2)	7 (3.7)	1 (0.5)
Abdominal distension	9 (9.9)	9 (9.9)	0 (0)	30 (15.8)	30 (15.8)	0 (0)
Anorexia	19 (20.9)	19 (20.9)	0 (0)	12 (6.3)	12 (6.3)	0 (0)
Ascites	0 (0)	0 (0)	0 (0)	1 (0.5)	1 (0.5)	0 (0)
Typhlitis	0 (0)	0 (0)	0 (0)	2 (1.1)	2 (1.1)	0 (0)
Nausea	3 (3.3)	3 (3.3)	0 (0)	7 (3.7)	7 (3.7)	0 (0)
Vomiting	4 (4.4)	3 (3.3)	1 (1.1)	4 (2.1)	4 (2.1)	0 (0)
**Hepatic funtion**						
Elevated ALP	40 (44.0)	40 (44.0)	0 (0)	100 (52.6)	99 (52.1)	1 (0.5)
Elevated ALT	91 (100)	70 (76.9)	21 (23.1)	89 (46.8)	85 (44.7)	4 (2.1)
Elevated TBIL	38 (41.8)	32 (35.2)	6 (6.6)	50 (26.3)	44 (23.2)	6 (3.2)
Hypoalbuminemia	54 (59.3)	54 (59.3)	0 (0)	120 (63.2)	120 (63.2)	0 (0)
**Renal/Genitourinary**						
Elevated CRE	16 (17.6)	16 (17.6)	0 (0)	32 (16.8)	30 (15.8)	2 (1.1)

ALP, alkaline phosphatase; ALT, alanine aminotransferase; TBIL, total bilirubin; CRE, creatinine. TACE, transarterial chemoembolization; HAIC, hepatic arterial infusion chemotherapy;
